# 奥妥珠单抗、糖皮质激素为基础的联合方案治疗复发性免疫性血栓性血小板减少性紫癜4例

**DOI:** 10.3760/cma.j.cn121090-20241107-00437

**Published:** 2025-01

**Authors:** 海菊 何, 云 李, 竑 田, 晓燕 徐, 健 苏, 鑫鑫 葛, 德沛 吴, 自强 余, 杰 殷

**Affiliations:** 苏州大学附属第一医院血液内科，江苏省血液研究所，国家血液系统疾病临床医学研究中心，国家卫生健康委员会血栓与止血重点实验室，血液学协同创新中心，苏州 215006 Jiangsu Institute of Hematology, National Clinical Research Center for Hematologic Diseases, NHC Key Laboratory of Thrombosis and Hemostasis, The First Affiliated Hospital of Soochow University, Collaborative Innovation Center of Hematology, Soochow Universtiy, Suzhou 215006, China

**Keywords:** 免疫性血栓性血小板减少性紫癜, 复发, 奥妥珠单抗, 临床缓解, Immune thrombotic thrombocytopenic purpura, Relapse, Obinutuzumab, Clinical remission

## Abstract

**目的:**

探索奥妥珠单抗、糖皮质激素为基础的联合方案在复发性免疫性血栓性血小板减少性紫癜（iTTP）患者中的疗效与安全性。

**方法:**

回顾性分析2023年1月1日至2024年6月30日苏州大学附属第一医院血液内科收治的4例经过奥妥珠单抗、糖皮质激素为基础联合治疗的复发性iTTP患者。

**结果:**

4例复发iTTP患者均接受过利妥昔单抗和硼替佐米治疗，3例存在其他自身抗体。4例患者经过奥妥珠单抗和糖皮质激素为基础的联合治疗后均获得临床缓解，ADAMTS13活性恢复正常，抑制物转阴。中位随访11（3～17）个月，4例患者均为持续缓解状态。所有患者治疗及随访期间未出现严重不良反应。

**结论:**

奥妥珠单抗、糖皮质激素为基础的联合方案治疗复发性iTTP具有良好的疗效和安全性。

## Abstract (en)

**Objective:**

To evaluate the efficacy and safety of obinutuzumab combined with glucocorticoid-based therapy in patients with relapsed immune thrombotic thrombocytopenic purpura (iTTP).

**Methods:**

This study analyzed the efficacy and adverse reactions of four patients with relapsed iTTP who were treated with a combination of obinutuzumab and glucocorticoids to assess the effectiveness and safety of the treatment.

**Results:**

All four patients had a history of multiple relapses and had previously undergone treatment with rituximab and bortezomib. Three patients exhibited additional autoantibodies. Following the combined therapy, all patients achieved clinical remission, with ADAMTS13 activity returning to normal levels and inhibitors testing negative. During a median follow-up period of 11 months (range: 3–17 months), all patients maintained sustained remission. No severe adverse events were reported during treatment or follow-up.

**Conclusion:**

The combination of obinutuzumab and glucocorticoid-based therapy is effective and safe for treating relapsed iTTP.

血栓性血小板减少性紫癜（TTP）是一种罕见且危重的血栓性微血管病，发病机制主要是由各种原因导致的血管性血友病因子（VWF）裂解酶ADAMTS13活性降低，血浆中大分子量VWF不能被降解，进一步诱发血小板的黏附、激活和聚集，从而形成广泛的微血栓。临床特征包括微血管病性溶血性贫血、消耗性血小板减少和靶器官损害。多数的TTP是由于免疫因素介导产生ADAMTS13酶的自身抗体，导致ADAMTS13酶获得性缺乏，即免疫性血栓性血小板减少性紫癜（iTTP）；少部分患者是由于ADAMTS13基因突变引起的遗传性缺乏。

对于iTTP，目前一线的治疗包括糖皮质激素、血浆置换、利妥昔单抗和卡普赛珠单抗。随着这些治疗方案的应用，难治/复发率已经由40％降至10％以下[1]。但是对于复发或难治的iTTP，尚无明确的推荐治疗方案。我中心在内的一些中心使用蛋白酶体抑制剂硼替佐米治疗复发/难治的iTTP，获得较好的疗效[2]，但仍存在少部分患者不能耐受、治疗无反应或缓解后再复发等问题[2]。奥妥珠单抗是人源化糖基化的Ⅱ型CD20单抗，相较于Ⅰ型CD20单抗（如利妥昔单抗），其直接细胞杀伤作用（DCD）作用更强，因Fc端经过糖基化修饰，与效应细胞结合增强，从而增强抗体依赖的细胞毒作用（ADCC）和抗体依赖的细胞吞噬作用（ADCP）。在治疗慢性淋巴细胞白血病（CLL）、滤泡淋巴瘤（FL）以及边缘区淋巴瘤（MZL）时，奥妥珠单抗相较于利妥昔单抗诱导缓解率更高，缓解程度更深，且无进展生存期更长[3]–[4]。然而奥妥珠单抗在自身免疫性疾病中的应用值得进一步探索，目前有个别临床试验及少数个案报道，取得了较好的疗效[5]–[11]。我们近期应用奥妥珠单抗、糖皮质激素为基础的联合方案治疗4例复发性iTTP患者并获得较好的临床疗效。

## 病例与方法

一、病例

本研究为回顾性研究，纳入自2023年1月1日至2024年6月30日在我中心接受奥妥珠单抗、糖皮质激素为基础联合方案治疗的4例复发性iTTP患者。所有患者均符合国际血栓与止血学会TTP诊断指南[12]及《血栓性血小板减少性紫癜诊断与治疗中国指南（2022年版）》[13]中iTTP的诊断标准。iTTP临床复发：在获得临床缓解后，再次出现血小板计数<100×10^9^/L且ADAMTS13活性<10％，伴或不伴脏器缺血损伤临床证据；ADAMTS13复发：ADAMTS13活性为0％、抑制物检测阳性，不伴血小板减少、贫血、器官损伤等临床表现。本研究中ADAMTS13活性的测定采用改良残余胶原结合实验。

二、治疗方案

奥妥珠单抗1 000 mg第1、15天各1次；甲泼尼龙80 mg/d，达到临床缓解后逐渐减停。奥妥珠单抗输注前及过程中常规给予预防过敏措施。临床复发急性期患者加用血浆置换，血小板计数恢复正常后停止，血浆受限时使用蛋白A免疫吸附清除抑制物。本组患者中2例进行了血浆置换，1例接受蛋白A免疫吸附（PAI）治疗，2例患者因存在风湿性疾病自身抗体加用硫酸羟氯喹0.4 g/d口服。

三、疗效评估和随访

疗效评估标准参照《血栓性血小板减少性紫癜诊断与治疗中国指南（2022年版）》。通过查阅患者住院/门诊病历及电话联系方式进行随访。末次随访时间为2024年9月30日。

## 结果

一、临床特征

本组4例患者中，男1例，女3例，中位年龄为30（24～70）岁。中位复发次数3（2～7）次，中位病程95（47～137）个月，既往使用免疫抑制剂种类中位数为2（2～4）种，均包括利妥昔单抗及硼替佐米。利妥昔单抗剂量以标准方案为主；硼替佐米1.3 mg/m^2^皮下注射，第1、4、8、11天，28 d为1个疗程，共2个疗程。例1前期免疫抑制治疗不充分，除糖皮质激素和血浆置换外，仅使用过环孢素A，缓解时间短且多次复发；近期复发后使用硼替佐米、利妥昔单抗治疗，缓解时间延长，但仍再次复发，病程中共复发7次。患者具体临床资料见表1。3例患者因皮肤瘀点瘀斑或牙龈出血就诊，完善检查后确诊iTTP临床复发，1例在定期随访中发现ADAMTS13复发。4例患者中有1例伴有系统性红斑狼疮，1例合并未分化结缔组织病。

二、实验室检查

3例临床复发患者均有血小板减少，2例患者有贫血，3例均有乳酸脱氢酶（LDH）升高，肾功能均正常。1例患者随访过程中发生ADAMTS13活性降低，抑制物转为阳性，不伴发热、出血、神经系统异常等临床表现，PLT、HGB、LDH、肌酐均在正常范围内，诊断为ADAMTS13复发。4例患者中3例检出抗核抗体、抗心磷脂抗体、甲状腺相关抗体、红细胞抗体等其他自身抗体，1例临床复发患者头颅磁共振成像（MRI）示亚急性脑梗死及微出血灶。详见表1。

**表1:** 4例复发性免疫性血栓性血小板减少性紫癜患者的临床特征及实验室检查

例号	年龄（岁）	性别	病程（月）	伴随疾病	PLT（×10^9^/L）	HGB（g/L）	LDH（U/L）	Cr（µmol/L）	ADAMTS13（％）	ADAMTS13抑制物	其他自身抗体
1	24	女	131	无	15	105	681.7	62.0	0	阳性	TgAb
2	70	女	59	系统性红斑狼疮	6	104	432.4	62.2	0	阳性	ANA, anti-SSA/RO52KD, anti-Histone，anti-ds-DNA, anti-SSB, TgAb, TPOAb
3	34	女	47	结缔组织病	30	118	293.7	50.1	0	阳性	ANA, anti-U1-snRNP, aCL
4	26	男	137	无	152	145	231.0	49.0	0	阳性	–

**注** LDH：乳酸脱氢酶；Cr：肌酐；Tg-Ab：甲状腺球蛋白抗体；ANA：抗核抗体；SSA/RO52KD：抗干燥综合征A抗体（RO52KD）；anti-histones：抗组蛋白抗体；anti-ds-DNA：抗双链DNA抗体；anti-SSB：抗干燥综合征B抗体；TPO-Ab：甲状腺过氧化物酶抗体；anti-U1-snRNP：抗U1核糖蛋白抗体；aCL：抗心磷脂抗体

三、治疗方案和疗效评估

所有患者入院即开始甲泼尼龙80 mg/d治疗，3例临床复发患者中2例在急性期进行了血浆置换，另外1例因血浆供应受限，进行了PAI治疗。4例患者中，1例（例1）因经费原因仅使用1剂奥妥珠单抗1 000 mg，其余3例均接受2剂奥妥珠单抗1 000 mg治疗一个疗程（第1、15天各1剂）。联合治疗后，4例患者均获得临床缓解，ADAMTS13活性恢复正常、抑制物转阴，获得临床缓解的中位时间为10（3～21）d（表2）。

**表2:** 4例复发性免疫性血栓性血小板减少性紫癜患者的复发、治疗及随访结果

例号	既往治疗	复发次数	本次复发	奥妥珠用量	不良反应	伴随治疗	治疗反应	随访时间（月）	随访截止状态	随访截止用药
1	糖皮质激素，CsA，TPE，硼替佐米，雷帕霉素，RTX	7	临床复发	1 000 mg×1	无	甲泼尼龙，TPE	CR	17	CR	泼尼松10 mg/d
2	糖皮质激素，TPE，RTX，硼替佐米	2	临床复发	1 000 mg×2	2级IRR，上呼吸道感染	甲泼尼龙，TPE，羟氯喹	CR	14	CR	羟氯喹0.4 g/d
3	糖皮质激素，TPE，RTX，硼替佐米	2	临床复发	1 000 mg×2	无	甲泼尼龙，蛋白A免疫吸附，羟氯喹	CR	8	CR	羟氯喹0.4 g/d
4	糖皮质激素，TPE，RTX，硼替佐米	4	ADAMTS13复发	1 000 mg×2	无	甲泼尼龙	CR	3	CR	甲泼尼龙4 mg/d

**注** TPE：治疗性血浆置换；RTX：利妥昔单抗；CR：临床缓解；IRR：输液相关反应。例2使用RTX过程中出现严重过敏反应

四、不良反应及复发

例2在初次使用奥妥珠单抗过程中出现2级输液相关反应（IRR），表现为血压短暂下降，予调整输注速度后血压恢复。该例患者在既往利妥昔单抗治疗过程中曾出现严重的过敏反应，表现为皮疹、呼吸困难，血压下降，需要血管活性药物维持血压。其余3例患者在奥妥珠单抗使用期间均未发生过敏反应。4例患者在使用后均未出现中性粒细胞减少、血小板减少。例2患者分别在用药后5、12个月发生上呼吸道感染，口服抗生素治疗后好转。至2024年9月30日随访截止，4例患者中位随访11（3～17）个月，均处于持续缓解状态（表2），2例患者已减停糖皮质激素，2例患者小剂量糖皮质激素维持中。

## 讨论

TTP是罕见的危重症，难治复发的iTTP目前尚无标准的治疗方案。本研究中我们尝试了使用奥妥珠单抗治疗有多次复发、既往使用过利妥昔单抗和硼替佐米的iTTP，患者均获得缓解，随访至今仍未复发。

奥妥珠单抗获批应用于FL和CLL，在头对头的临床试验研究中，其疗效优于利妥昔单抗[3]。在风湿免疫系统疾病中，奥妥珠单抗应用仅有少量个案报道，包括膜性肾病、系统性红斑狼疮、IgG4相关性眼病、ANCA相关血管炎等，均获得良好的初步疗效[6]–[7],[10]–[11]。Robertz等[9]报道应用奥妥珠单抗治疗2例利妥昔单抗不耐受的iTTP患者，均获得持续缓解。一项英国多中心的回顾性研究总结了对利妥昔单抗不耐受iTTP患者应用其他CD20单抗（奥法木单抗和奥妥珠单抗）治疗，其中8例经奥妥珠单抗治疗后均获得缓解，随访中仅1例复发[8]。本研究中的iTTP患者均为多次复发，既往治疗过程中都使用过利妥昔单抗及硼替佐米，治疗难度大，药物选择有限。2例患者合并其他自身免疫性疾病，3例患者存在其他自身抗体。我们既往研究也发现，患者的这种自身免疫紊乱状态可能与iTTP的多次复发有关[2]。因此使用更强的B细胞清除治疗可能会使这部分患者获益。本研究中4例iTTP患者经奥妥珠单抗、糖皮质激素为基础的联合方案治疗，均获得持续缓解。

奥妥珠单抗是Ⅱ型CD20单抗，且Fc端经过了糖基化修饰。作为Ⅱ型CD20单抗，奥妥珠单抗和细胞表面CD20结合后可引起更强的直接细胞杀伤作用（DCD）；同时增强了同质黏附作用，使靶细胞黏附聚集，增强了免疫系统对其识别和杀伤。糖基化修饰后的Fc端能够增强效应细胞对靶细胞的识别和吞噬，从而引起更强的ADCC和ADCP作用；同时也能提高抗体本身在体内的稳定性，延长半衰期。而利妥昔单抗作为Ⅰ型CD20单抗，与CD20抗原结合使CD20抗原重新定位到脂筏中，其引起的DCD作用很弱，主要是能诱导补体依赖性细胞毒性（CDC）和抗体依赖性细胞毒性（ADCC）。但实际上，因为体内存在补体抗性因子或者补体耗竭，Ⅰ型抗CD20单克隆抗体激活CDC的能力在体内可能是有限的[14]。综上所述，奥妥珠单抗和利妥昔单抗作用有所不同，且奥妥珠单抗有更强的B细胞清除作用，这可能是本研究中患者使用利妥昔单抗后复发，继续使用奥妥珠单抗仍然有效的原因。其他一些研究也有类似的发现，比如在对利妥昔单抗反应不佳的狼疮肾炎患者中显示了很好的疗效，这可能跟奥妥珠单抗有更强的B细胞清除作用有关，可清除肾脏组织中未被利妥昔单抗清除的B淋巴细胞，因而可能会达到更深的缓解[5],[7],[15]。治疗CLL和FL的临床研究中，奥妥珠单抗除了诱导缓解率更高以外，缓解程度也更深，表现为奥妥珠单抗治疗后缓解的病例中MRD阴性率较高[14],[16]。

奥妥珠单抗在治疗CLL和非霍奇金淋巴瘤（NHL）时最常见的不良反应是IRR[3]，而在治疗免疫相关疾病时，IRR发生率却明显降低[5],[7]–[11]。本研究中也仅1例发生Ⅱ级IRR。CD20单抗治疗CLL和NHL时IRR的发生主要考虑与B细胞清除引起的细胞因子释放有关，发生IRR的患者比未发生的患者释放更多的IL-8、IL-6和TNF-α，循环中B细胞负荷高的患者发生IRR的风险也更高[17]–[19]。而自身免疫相关疾病患者循环中并无显著增高的B细胞负荷，这可能是严重IRR发生率不高的原因[5]。本研究中例2患者在既往使用利妥昔单抗时出现严重的皮疹、呼吸困难、休克，考虑为Ⅰ型过敏反应。该患者使用奥妥珠单抗时，给予常规预防过敏措施，仅在初次输注时出现了Ⅱ级输液反应。一项体外研究发现，产生利妥昔单抗抗体的患者血清与奥妥珠单抗并无交叉过敏反应[20]。在两个关于不耐受利妥昔单抗的iTTP患者治疗报道中，患者对利妥昔单抗有严重的过敏反应（包括输注相关/过敏反应、血清病），转而使用Ⅱ型CD20单抗（奥法木单抗或奥妥珠单抗）后均能够完成治疗，28例次治疗中仅2例有轻微IRR[8]–[9]。一些不能耐受利妥昔单抗的血液系统恶性肿瘤患者转为使用奥妥珠单抗治疗，也基本能耐受[21]。因此，对利妥昔单抗有过敏反应的患者，奥妥珠单抗仍可选择使用。

除了过敏反应，感染也是CD20单抗常见的不良反应，奥妥珠单抗治疗B淋巴细胞恶性肿瘤时感染率有所增加[3]。B淋巴细胞恶性肿瘤患者本身体液免疫受损，奥妥珠单抗联合化疗治疗进一步抑制免疫并产生骨髓抑制，这可能是该类患者感染发生率高的原因。但奥妥珠单抗治疗自身免疫疾病，目前尚无重症感染的报道，一方面可能与病种相关，另一方面也不排除研究病例数有限的影响。但在本研究中，仅有1例患者在用药后用药后5个月和12个月各发生1次上呼吸道感染，口服抗感染药物后好转。今后需要开展更多大规模临床试验来评估奥妥珠单抗治疗自身免疫性疾病的安全性。

综上所述，本研究结果初步显示奥妥珠单抗、糖皮质激素为基础的联合方案治疗复发性iTTP可获得持续缓解且具有良好的安全性。本研究为回顾性研究且例数有限，奥妥珠单抗在复发性iTTP中的疗效尚需要前瞻性随机对照研究进一步证实。
